# The Feature, Performance, and Prospect of Advanced Electrodes for Electroencephalogram

**DOI:** 10.3390/bios13010101

**Published:** 2023-01-06

**Authors:** Qing Liu, Liangtao Yang, Zhilin Zhang, Hui Yang, Yi Zhang, Jinglong Wu

**Affiliations:** Shenzhen Institute of Advanced Technology, Chinese Academy of Sciences, 1068 Xueyuan Avenue, Shenzhen 518055, China

**Keywords:** EEG, advanced electrodes, impedance, signal quality, artifacts, biofeatures, application scenarios

## Abstract

Recently, advanced electrodes have been developed, such as semi-dry, dry contact, dry non-contact, and microneedle array electrodes. They can overcome the issues of wet electrodes and maintain high signal quality. However, the variations in these electrodes are still unclear and not explained, and there is still confusion regarding the feasibility of electrodes for different application scenarios. In this review, the physical features and electroencephalogram (EEG) signal performances of these advanced EEG electrodes are introduced in view of the differences in contact between the skin and electrodes. Specifically, contact features, biofeatures, impedance, signal quality, and artifacts are discussed. The application scenarios and prospects of different types of EEG electrodes are also elucidated.

## 1. Introduction

An electroencephalogram (EEG) is a graph of the difference in voltage between brain scalp locations over time. In medicine, EEG signals can not only provide diagnostic evidence for brain diseases but also act as effective rehabilitation equipment to help restore the activities of patients [1,2,3,4]. In terms of engineering applications, utilizing the differences in the EEG signals of sensory, motor, and cognitive activities, EEG can be applied to control or realize projected activities with external devices [5]. Because the EEG signal is nonstationary and random [6], it usually presents low signal intensity and high noise with a typical amplitude of 10–100 µV [7]. Therefore, the development of advanced electrodes to obtain high-quality EEG signals is a very attractive but challenging research topic. The Ag/AgCl wet electrode is usually used with a conductive gel to reduce the electrode–skin impedance for high-quality EEG signals, which has been the gold standard for the real-life recording of brain potentials [8,9]. However, several issues remain to be addressed. First, skin preparation is mandatory for wet electrodes, such as hair cutting (in some cases), decontamination, and electrolyte coating, which are very time-consuming. Additionally, the conductive gel dehydrates and coagulates, leading to a reduction in signal quality after long-term recording. Moreover, it has been observed that gel causes allergic reactions and skin irritation in some patients [9,10]. For BCI, wet electrode-equipped systems not only require a long installation time but are also user-uncomfortable. It lacks the convenience of use, and continuous long-term EEG monitoring needs further investigation. In light of these concerns, designing advanced electrodes is highly desirable. Researchers have been searching for solutions to overcome the challenges of wet electrodes while ensuring good signal quality. Novel semi-dry electrodes, dry contact electrodes, dry non-contact electrodes, and microneedle array electrodes have been reported [11,12,13,14,15,16,17]. However, the configuration features and signal performance remain unclear and have not yet been compared. This review compares the electrode–skin contact performances of these types of EEG electrodes with Ag/AgCl wet electrodes. Combined with the requests of the users and the signal quality, the most suitable electrodes are considered to have the following characteristics: (1) low electrode–skin contact impedance, (2) excellent biocompatibility with good comfort, and (3) high signal quality and small artifacts.

Recently, Lopez et al. reviewed approaches for developing dry EEG electrodes for clinical and other applications [8]. In another report, our group also discussed the materials for dry EEG electrodes in terms of preparation and EEG signal properties [18]. The fabrication methods, mechanical properties, and bioelectrical signal recording of microneedle and fabric textile electrodes were also reviewed by Ren et al. [19] and Acar et al. [20]. Sun. reported the latest developments in capacitive biopotential measurements for electrophysiological signal acquisition [21]. Li et al. reviewed the development, evaluation methods, and practical design considerations of semi-dry electrodes [22]. These reports indicate that emerging gel-free electrodes have attracted considerable attention owing to their multifaceted advantages. However, previous studies have not covered all types of gel-free electrodes, and the variations in electrode types, features, and performances between different electrodes are not yet clear. Moreover, there is confusion regarding the feasibility of candidate electrodes for different application scenarios.

**Figure 1:**
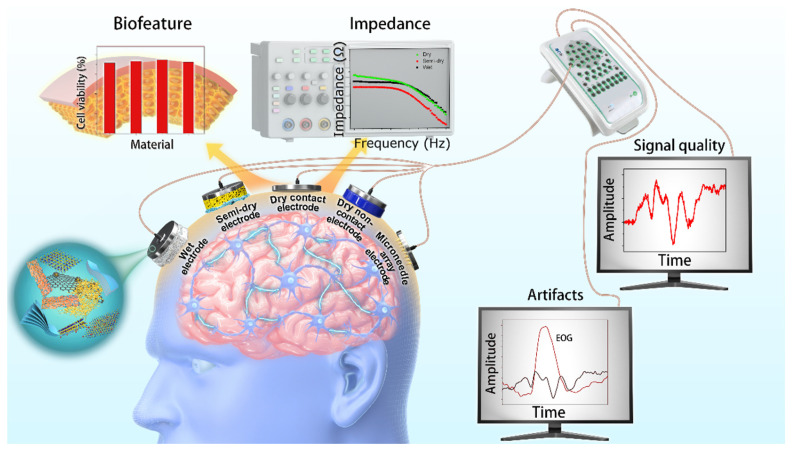
Illustration of various EEG electrodes in configuration, features, and performances.

In this review, Section 2 presents the features and performance of different types of EEG electrodes, including contact features, biofeatures, impedance, signal quality, and artifacts. Figure 1 schematically illustrates the main aspects of this report with respect to configuration, features, and performance. The contact features and biofeatures between the electrode and the skin of five different types of electrodes are described. Subsequently, the EEG signal performance obtained from different types of electrodes is presented and discussed. The application scenarios of different electrodes are summarized according to their physical features and EEG performance. This report elucidates the variations in advanced EEG electrodes not only in physical features and EEG performances but also in application scenarios.

## 2. Feature and Performance

### 2.1. Contact Feature

**Figure 2:**
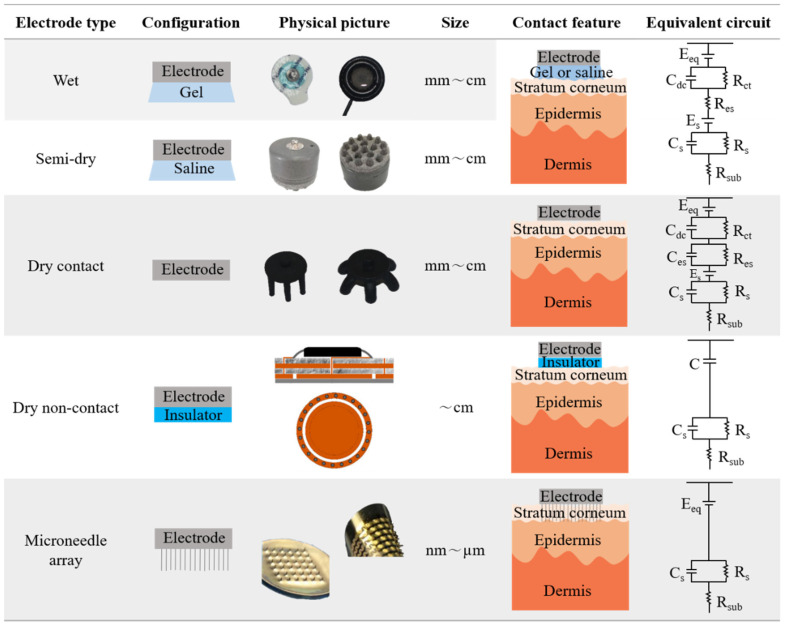
Summary of electrode configuration, electrode-skin contact features, and electrical equivalent circuits of different types of EEG electrodes. (The electrode schematic diagram and equivalent circuit diagram are referenced from the literature [9,22]. Photographs of wet and dry electrodes are provided by grael, semi-dry electrodes are from [23], dry non-contact electrodes are from [24], and microneedle array electrodes are from [25,26]).

The contact feature is defined here to explain the characteristics of the contact between the electrode and the skin of different types of electrodes. When the electrode was placed on the skin, the contact varied owing to configuration differences, leading to a difference in electrical activity at the interface. An equivalent circuit is also used to explain the different electrical activities, as displayed in Figure 2. In particular, these electrodes are easily divided into wet electrodes and semi-dry electrodes (electrolyte contact), dry contact electrodes (surface contact), dry non-contact electrodes (non-contact), and microneedle array electrodes (invasive contact), according to the difference in contact on the epidermis.

The configuration of the wet electrode, which is most frequently used in clinics and laboratory experiments, consists of a disc and gel. The disc is usually made or coated with Ag/AgCl. This electrode is typically used in combination with gel electrolytes. Therefore, the disc did not touch the skin surface directly. The gel was located between the electrode and the skin. When the electrolyte is a small amount of flowing liquid, such as water, saline, or phosphate-buffered saline (PBS), the electrolyte can be easily removed from the skin surface. Therefore, in some reports, electrodes using a small amount of water-fluid electrolytes are called semi-dry electrodes or quasi-dry electrodes [27]. This is similar to a wet electrode, in which the water-fluid electrolyte directly contacts the skin. Compared to electrolytic electrodes (wet and semi-dry electrodes), dry contact electrodes do not require a wet electrolyte between the disc and skin. The dry contact electrode touched the skin directly. It consisted of only a disc. These discs are typically composed of metal or conductive polymer materials. It is currently the most promising candidate for many applications. Dry non-contact electrodes have a dielectric material added between the disc and skin. This dielectric material can be textured (cloth), oxides, ceramics, plastics, or organics [28]. Dielectric materials usually touch the skin surface, and the electrode disc is placed on the dielectric materials. The electrode and skin form two electrical fields, that is, capacitance, which creates a potential that can be detected by the EEG system. Since the electrical resistance of the stratum corneum is much higher than that of the epidermis [9], the microneedle array electrode was developed to penetrate the highly resistant stratum corneum to improve the signal quality. It consists of a substrate, and a needle array is built on it. The length of the needles is usually in µm, and the diameter ranges from nm to µm. The needle penetrated the skin surface and contacted the epidermis. However, it is invasive compared with other electrodes.

An equivalent circuit is a model comprising electrical elements. This model was established based on the electrical activity between the electrode and skin. It is extensively used to understand electrical activity. The electrical current is generated in cells or neurons. It flows to the dermis and intercellularly, leading to internal body resistance, which is called R_sub_. The interface between the epidermis and dermis is equivalent to the parallel capacitance and resistance, namely, C_s_ and R_s_ [9,19,22,28,29] The gel of the wet electrode exists between the skin and the electrode. Thus, the electrode–electrolyte interface (EEI) and electrolyte–skin interface (ESI) were formed. The wet electrode is characterized by an electrode potential difference E_eq_, double-layer capacitance, and parallel and series resistances, as shown in Figure 2 [29,30,31,32]. For the semi-dry electrode, the water-fluid electrolyte in the electrode cavity is released to the scalp under pressure to establish a relatively stable electrode–scalp interface [33]. Thus, the equivalent circuit is the same as that of a wet electrode [34]. Dry contact electrodes were tightly placed on the scalp surface. Compared with wet electrodes, the equivalent circuit model adds capacitance in parallel with resistance. A dry non-contact electrode (also called a capacitive electrode) means that the electrode does not contact the skin. It is coupled with the skin in a non-contact manner through air, clothes, hair, or other electronic materials. Therefore, the element of the dielectric materials can be described as capacitance C, and the remaining parts are the same as those of the wet electrodes. A microneedle array electrode is an invasive electrode whose microneedles penetrate the stratum corneum of the skin. It is clear that there is only an electrode–skin interface characterized by an electrode potential difference E_eq_. An equivalent circuit diagram is presented in Figure 2. From the equivalent circuit diagrams of the respective electrodes, it can be seen that semi-dry electrodes are the closest class of electrodes to commercial wet electrodes, which also provides a promising and effective alternative electrode, however, its performance needs to be evaluated in many aspects, as described below. The microneedle array electrode is the only EEG electrode that penetrates the stratum corneum to the dermis, showing fewer circuit-fitting devices between the dermis and the electrode, which shows a lower impedance, but it is especially necessary to pay attention to the impact of puncture on human comfort.

### 2.2. Biofeature

The biofeatures indicate the biocompatibility and comfort of the electrode to the subjects. Biocompatibility reflects the compatibility of a device with a biological system (not with any local or systemic response from a living system or tissue). In other words, the EEG electrode did not damage the subjects. The International Organization for Standardization (ISO) has established a standard for identifying medical products (ISO 10993 standard). Therefore, the biocompatibility evaluation of EEG electrodes should be composed of an in vitro cytotoxicity test (based on ISO 10993-5) and a skin irritation test (based on ISO 10993-10).

In vitro cytotoxicity tests have general applicability and are widely used in the evaluation of various devices and materials. This review does not specify a single test, but rather defines a test protocol that requires a decision through a series of steps, which help select the most appropriate tests. According to ISO 10993-5, there are three types of tests: extraction tests, direct contact tests, and indirect contact tests. The choice of one or more of these categories depends on the properties of the sample, the potential site of use, and the nature of the use. Then, the details of the preparation of the sample to be tested, the preparation of the cultured cells, and the manner in which the cells are exposed to the sample or its extracts are determined. The presence and extent of cytotoxic effects were assessed at the end of the exposure period. The numerous methods used in cytotoxicity assays and the end points of the measurements can be grouped into the following assessment categories: 1. Cell damage was evaluated by morphological means; 2. Measurement of cell damage; 3. Measurement of cell growth; 4. Measure specific aspects of cell metabolism. In each of these four categories, there are several ways to produce results. The investigator should be aware of the test categories and the categories for which a particular technique fits so that other results can be compared with similar devices or materials at both the intra- and interlaboratory levels [35].

Skin irritation tests assess possible exposure hazards from chemicals released by medical devices that may produce skin sensitization. Some of the materials contained in existing medical devices have been tested, and their skin sensitization potential, primarily pure chemicals, has been documented. However, there are other materials and their chemical compositions that have not been tested, especially some emerging composites, which may cause adverse effects when they come into contact with human tissue. It is therefore incumbent on the producer to assess the potential adverse effects of each device prior to application. Traditionally, small animal testing is performed before testing on humans to help predict human responses. In order to reduce the number of animals used, where appropriate, initial screening using in vitro methods prior to animal testing is encouraged, with a step-by-step approach where test results are reviewed and analyzed at each stage [36].

Comfort refers to the comprehensive evaluation of people’s satisfaction with the objective environment from both physical and psychological aspects. Affected by the conditions, the comfort level will show different results owing to differences in individual feelings. Comfort evaluation is usually conducted by attaching an EEG electrode to the upper arm or other selected parts of the subject for a certain period of time. The participants were then required to complete a questionnaire related to comfort [37]. There are two main forms of survey questionnaires, one of which divides comfort into four levels: (i) comfort with mild pressure but no pain; (ii) mild pain; (iii) painful but acceptable pain; and (iv) obvious discomfort [38,39]. The other divided the comfort level questionnaire between 1 and 10. 1 represents the total comfort level and 10 represents the maximum imaginable pain [40,41]. The subjects reported that the Ag/AgCl wet electrode caused discomfort during the testing process with the application and removal of the gel [42]. Semi-dry electrodes are also in contact with electrolytes, and it is more acceptable for subjects to use water-fluid electrolytes, such as water, physiological saline, NaCl solution, and PBS.

It should be noted that in order to ensure good contact between the electrodes and the scalp, the applied pressure should be in a comfortable range, this is equally important for semi-dry and dry electrodes [37,41,43,44,45,46]. Compared to dry electrodes that are directly attached to the scalp without electrolytes, non-contact electrodes have a dielectric layer between the electrode and the skin, and the dielectrics are used in daily life [11,47,48,49,50]. Comfort is usually much better than wet, semi-dry, or dry contact electrodes. Comfort tests are rarely conducted for microneedle array electrodes [38], instead of insertion experiments [51,52]. Penetration always causes discomfort to subjects. At present, comfort is becoming increasingly important for the experience of subjects, especially for long-term signal recording.

### 2.3. Impedance

**Figure 3:**
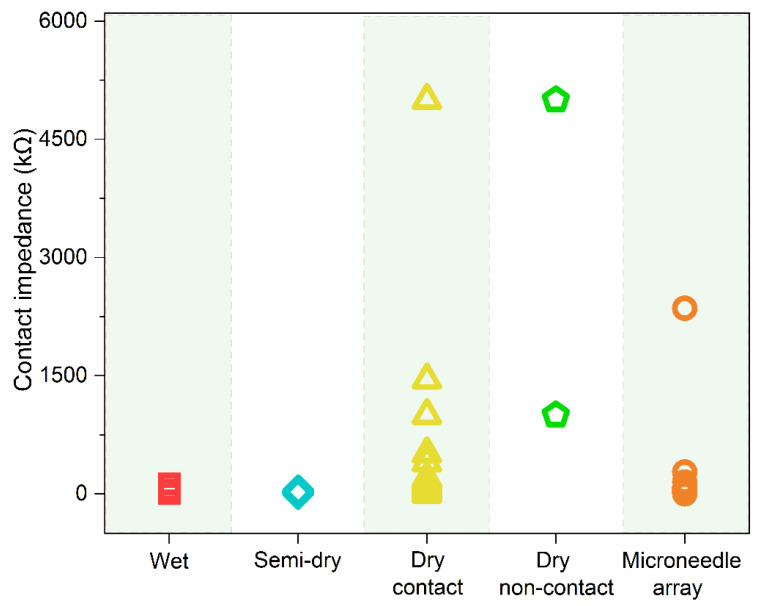
The electrode-skin contact impedance of wet electrodes (Data derived from [52,53,54,55,56,57,58].), semi-dry electrodes (Data derived from [23,37,45,59,61,62,63,64,65,66,67]), dry contact electrodes (Data derived from [39,40,41,44,46,53,55,56,69,70,71,72,73,74,75,76,77,78,79,80,81,82,83,84,85,86,87,88,89,90,91,92,93,94,95,96,97,98]), non-contact electrodes (Data derived from [11,47]) and microneedle array electrodes (Data derived from [25,38,51,52,54,57,58,99,100,101,102,103,104,105,106,107,108,109,110,111,112,113]). Details are in Appendix A Table A2, Table A3, Table A4 and Table A5.

Here, impedance indicates the contact impedance between the EEG electrodes and the scalp. Because the gel can significantly improve the electrical properties between the skin and electrodes, Ag/AgCl wet electrodes have the advantages of low impedance, stability, high signal-to-noise ratio (SNR), and reliable signal. Commercial EEG products require an electrode-skin impedance below 5 kΩ for wet electrodes, while it ranges from 2.8–130 kΩ (Figure 3) during practical experiments [52,53,54,55,56,57,58]. Recently, an increasing number of scientists have turned their attention to another electrolytic electrode, the semi-dry electrode [22]. Instead, they choose only small amounts of electrolytes that show excellent biocompatibility, such as water [59,60], physiological saline [61,62,63,64,65], NaCl solution [23,37,66,67], PBS [37], and hydrating agent [27]. They reported contact impedance of these semi-dry electrodes is between 1.5 and 61.3 kΩ [23,37,45,59,61,62,63,64,65,66,67] (Figure 3, Appendix A Table A2 for details). Another study by our group showed that the impedance of semi-dry electrodes on human arm skin was lower than that of wet and dry electrodes, which was attributed to the difference in charge transfer resistance at the interface between the electrode and skin [68]. Dry contact electrodes usually exhibit higher impedance than invasive electrodes. The impedance value was in the range of 2.5 kΩ–5.0 MΩ [39,40,41,44,46,53,55,56,69,70,71,72,73,74,75,76,77,78,79,80,81,82,83,84,85,86,87,88,89,90,91,92,93,94,95,96,97,98] (Figure 3, Appendix A Table A4 for details). As there is no need to apply a gel during application, it can be used directly on the scalp. They are convenient, and no complicated skin preparation is required; therefore, they are suitable for use in emergency situations, such as healthcare and monitoring exercises [9]. Dry non-contact electrodes have the highest impedance between 1 and 5 MΩ (Appendix A Table A3 for details). Microneedle array electrodes can effectively remove the influence of the insulating stratum corneum, leading to a lower impedance (1.181 kΩ–2.357 MΩ) (Figure 3, Appendix A Table A5 for details) [25,38,51,52,54,57,58,99,100,101,102,103,104,105,106,107,108,109,110,111,112,113]. They have attracted much attention not only in EEG monitoring but also in other electrophysiological signal detections, such as electrocardiogram (ECG) and electromyogram (EMG).

In addition to the type of contact difference between the skin and electrode, the electrode configuration and applied pressure also affect the contact impedance between the skin and electrode. For example, Wang et al. conducted contact impedance experiments using 13-pin and 21-pin flexible dry electrodes made of polydimethylsiloxane (PDMS). The results showed that the 21-pin electrode had lower contact impedance. This was because the 21-pin electrode increased the contact area between the skin and electrode [91]. Arai et al. developed a polymer-based microneedle array electrode. When the electrode is placed naturally on the skin without an external force, the electrode–skin contact impedance is greater than 200 kΩ. When a force greater than 8 N was applied, the contact impedance was less than 10 kΩ [52]. Therefore, the force and contact area are two factors that affect contact impedance.

### 2.4. Signal Quality

**Figure 4:**
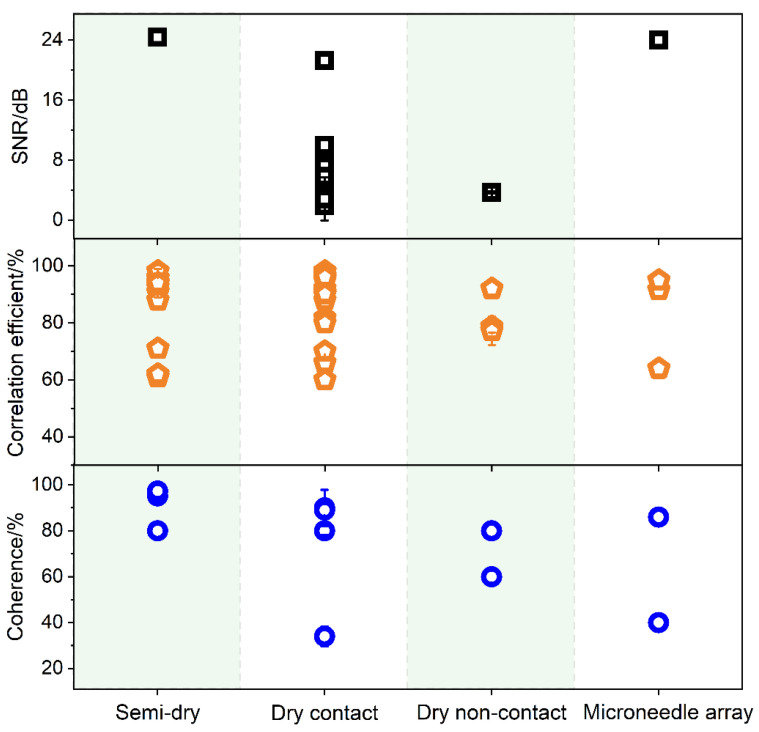
The SNR (black square), signal correlation (yellow diamond), and coherence (blue dots) of semi-dry electrodes (Data derived from [23,27,45,59,63,64,65,66]), dry contact electrodes (Data derived from [39,44,46,53,55,70,72,73,75,76,81,83,85,86,88,89,91,92,94,96,98,116,117,118,119,120,121]), dry non-contact electrodes (Data derived from [11,47,48]) and microneedle array electrodes (Data derived from [57,58,102]), details are in Appendix A Table A2, Table A3, Table A4 and Table A5.

The electrical signal indicates the spontaneous bioelectric activity of the brain detected by the electrode. The EEG signal is transmitted from the cerebral cortex through the skull and cerebrospinal fluid to the scalp; therefore, the EEG signal is weak [6]. To monitor high-quality signals, EEG electrodes must have excellent signal acquisition ability. To evaluate the signal quality of EEG electrodes, one factor is the SNR. It is defined as the ratio of signal power to noise power, which is used to compare the strength of the desired signal with the strength of the background noise [114]. SNR is defined by Appendix A Table A1 Equation (A1). As early as 1996, Miguel Angel Guevara and María Corsi-Cabrera used correlation and coherence to evaluate the quality of EEG signals [114]. Correlation (r) and coherence (Coh) are considered equivalent to the evaluation of the similarity degree between two signals [5,115]. The correlation is in the time domain, whereas the coherence is in the frequency domain. The correlation function (*r*) at each given frequency x is defined by Appendix A Table A1 Equation (A2), and the coherence function (Coh) at each given frequency x is defined by Appendix A Table A1 Equation (A3). Serdijn et al. compared the SNR, correlation, and coherence to evaluate the signal quality between wet and dry EEG electrodes [98]. Figure 4 shows a scatter graph of the SNR, correlation, and coherence of different types of reported electrodes.

Ag/AgCl wet electrodes with excellent electrical performances can provide accurate and stable EEG signals. It has been widely commercialized and is regarded as a standard clinical electrode. It was also used as a reference electrode to evaluate newly developed electrodes. Therefore, the signal quality of the developed EEG electrodes is usually compared with that of the Ag/AgCl wet electrode. In most reports, alpha rhythms modulated by opening and closing the eyes are often used to evaluate the signal quality of EEG electrodes [50,59,62,73,104]. The SNR, correlation, and coherence data for the semi-dry electrodes are shown in Figure 4. The result published in 2016 showed that the SNR of the semi-dry electrode was approximately 24.4 dB. As a comparison, the Ag/AgCl electrode is 24.9 dB, which is comparable to the commercial Ag/AgCl electrode [63]. The values of correlation between signals obtained by the semi-dry electrodes and Ag/AgCl wet electrode were all greater than 60%, and the highest value reached 98%. This shows that the waveform is consistent with that of the Ag/AgCl electrode. The coherence is between 80% and 97%, which shows that the signals recorded by the semi-dry and Ag/AgCl electrode exhibit a high degree of stability and consistency in frequency. The SNR of the dry contact electrodes varies from 3 to 24 dB, which is mainly due to the diversification of the electrode materials and structural designs. As a result, the performance of the electrodes varied. The correlation also ranged from 60% to 98%, and the coherence ranged from 34% to 90%. There are few reports on dry non-contact EEG electrodes. The SNR value of the dry non-contact EEG electrodes reported in the two articles is 3.7 ± 0.17 dB and 1.8 ± 0.13 dB, respectively [11,47]. This is due to strong noise effects. The correlation and coherence were 78–92% and 60–80%, respectively. For the microneedle array electrodes, the SNR of the polymer-based candle-shaped electrodes was 24.0 dB. As a reference, the Ag/AgCl electrode is 27.1 dB under the same conditions, which also shows a higher ability to resist interference and noise. The values of correlation and coherence were 60–95% and 40–86%, respectively. This reflects the higher signal quality of the microneedle array electrodes compared to that of the wet electrode.

The EEG statistics discussed above are all in the resting state; however, EEG signals during exercise also need to be considered. Hua et al. studied EEGs recorded by a flexible multilayer semi-dry and wet electrode during jogging. The correlation between the two EEG signals in a motion test was 90.65%, which proved that the EEG acquisition performance of the semi-dry electrode under motion was similar to that of the wet electrode [23]. Shu et al. investigated the signal quality of textile dry contact electrodes during jogging. The correlation of EEG signals measured by the wet and textile electrodes under the same motion conditions was 95.6%, which proves that the signal capture ability of the textile electrode under motion is almost as good as that of the patch wet electrode [73]. Tăuţan et al. analyzed the signal quality of the flexible polymer pinned dry contact EEG electrode during exercise, including correlation (50%), coherence (24%), and SNR (1.9 dB) [88,98]. Wang et al. used an ANSYS motion simulation to study the signal quality of a microneedle array electrode made of parylene-based materials. The results indicated that the parylene-based microneedle electrode array could conform to the skin motion and help decrease motion artifacts [102]. In summary, gel-free and wet electrodes have a certain waveform similarity. They show a correlation of greater than 60%, coherence greater than 40%, and SNR in the range of 3–25 dB. The semi-dry electrode signal quality outperformed that of the other types in both the time and frequency domains. In terms of signal quality, semi-dry and dry contact electrodes are the most promising for EEG. Moreover, it should be noted that the results can only be compared descriptively owing to the substantial differences in the collection devices in terms of the electrode numbers, electrode configurations, and reference electrodes.

### 2.5. Artifacts

The EEG signals were acquired from the scalp. Signal amplitude was also reduced by the skull, stratum corneum, and hair. Its amplitude is in microvolts (≤200 μV), which is lower than that of other physiological signals, such as ECG and EMG. Thus, it is more susceptible to interference, and the signals produced by these interferences are artifacts. Artifacts are categorized into (1) physiological, (2) equipment, and (3) environmental artifacts [122]. Physiological artifacts are generated by body parts other than the brain. The most representative movements are eye movements and muscular, electrocardiographic, and respiratory activity. The equipment artifacts were caused by the EEG system. The environmental artifacts are due to the power of line interference, high-frequency noise interference, and motion artifacts. In clinical applications, nonphysical artifacts can be eliminated by correctly connecting electrodes, adjusting the state of the subjects, and recording in a controlled environment. For home-monitored healthcare systems, extraneous sources of noise may exist [5,6]. A recent review by Sweeney et al. [123] dealt with noncontrolled recording environments. However, physiological artifacts cannot be avoided. Normally, the metrics employed to express the energy of the signal compared to the energy of the artifacts are the SNR [124] and the signal-to-artifact ratio (SAR) [122]. SAR is an energy ratio equivalent to the well-known SNR, but with only physiological artifacts as sources of contamination. Sergio et al. [125] calculated the SAR for each EEG channel to evaluate the extent of ocular contamination on the scalp. The index is defined as the ratio between the total power of the true cerebral component (EEG source) and the total power of the true electrooculogram (EOG) component (EOG source: corresponding to the propagation of both vertical and horizontal ocular activity to that channel), as shown in Appendix A Table A1 Equation (A4). Many computational studies have been conducted on artifact removal to improve SAR [122,126]. Hanna et al. investigated SAR enhancement by projecting principal components (PCs) [127]. The SAR was increased from 0.011 ± 0.002 dB to 0.47 ± 0.11 dB after removing 30 PCs. Saleha et al. explored SAR with two commonly used methods, Discrete Wavelet Transform (DWT) and Stationary Wavelet Transform (SWT), combined with Universal Threshold (UT) and Statistical Threshold (ST) [128]. The SWT + ST approach performs the best SAR of 2.33 ± 0.86 dB compared to the other combinations. Recently, Gabriel et al. analyzed EEG data using Schrödinger filtering, median filtering, amplitude thresholding, and wavelet denoising [129]. The SAR scores indicate that Schrödinger filtering outperforms the other three techniques. To the best of our knowledge, artifacts can be effectively eliminated or reduced by constructing appropriate EEG electrodes, including dry contact electrodes [44,130,131,132], dry non-contact electrodes [47,48], and active electrodes [133,134], especially intracranial electrodes [135,136,137] or microneedle array electrodes [7,110,138,139]. In addition, artifacts can be eliminated or reduced by combining them with other techniques such as functional magnetic resonance imaging (fMRI) [140,141], transcranial magnetic stimulation (TMS) [142], and near-infrared spectroscopy (NIRS) [143].

## 3. Conclusions and Prospects

The EEG electrodes were reviewed in terms of their features and performances. In the contact feature, the wet, semi-dry, and dry contact electrodes were all surface contact electrodes. The non-contact electrodes were surface non-contact electrodes, whereas the microneedle array electrodes were penetrating electrodes. In particular, the microneedle array electrode exhibited the lowest circuit component consumption, which indicated the lowest theoretical resistance. The semi-dry and wet electrodes showed similar contact impedances compared to the surface dry electrode owing to the presence of the electrolyte. Dry non-contact electrodes exhibit the largest resistance among all electrodes owing to the presence of capacitance. Dry contact and dry non-contact electrodes are the preferred electrodes for comfort both by the subjects and users, owing to their freedom from gel and skin intrusion. The contact impedance of the dry contact electrodes was much higher than those of the wet, semi-dry, and microneedle array electrodes. The impedance of the dry non-contact electrode was not comparable to that of the other electrodes owing to the lack of contact. However, there is evidence that the contact impedance can be tuned by adjusting the contact area and the applied pressure. The biofeatures of the different electrodes are demonstrated through biocompatibility and comfort. The coherence and SNR of the semi-dry electrode were better than those of other types of electrodes. This correlation is comparable to that of other gel-free electrodes. The artifacts can be effectively removed by optimizing the electrode and algorithm during EEG data analysis so that the SAR can be improved.

The presence of gel makes the impedance of wet electrodes drop rapidly, which has obvious advantages in laboratory research and medical diagnosis applications, especially in the field of cognitive neuroscience and medical diagnosis or treatment. Gel-free electrodes for EEG signal measurement, particularly semi-dry and dry contact electrodes, are comparable to traditional Ag/AgCl wet electrodes in terms of performance, convenience, and user comfort. The semi-dry electrode combines the advantages of wet and dry electrodes and exhibits excellent signal-recording performance. Although the electrode structure can be reasonably designed to ensure the continuous supply of electrolytes, the impact of electrolyte drying cannot be completely solved because the container size of the electrode is certain. Thus, semi-dry electrodes are suitable for short-term (generally several hours) monitoring. The microneedle array electrode penetrates the skin surface with low impedance and high signal quality, which is suitable for short-term monitoring with high requirements for signal quality. Dry contact electrodes and dry non-contact electrodes have the advantages of long-term monitoring and are comfortable for users to wear, which is feasible for wireless wearable electrode applications (e.g., brain–computer interfaces).

**Figure 5:**
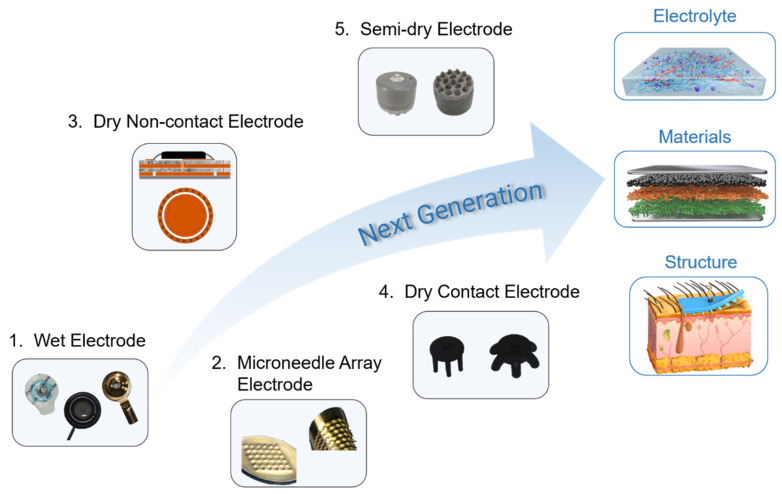
Existing and future trends electrodes.

Due to the simplex liquid or solid phase, the above five types of EEG electrodes with their interface have insufficient dynamic adaptability to hair and scalp. In order to maintain the stable and effective contact between the electrode and scalp for long-term EEG recording, the future EEG electrodes should be improved from the electrolyte phase, electrode material, and electrode structure (as shown in Figure 5). By changing the phase state, the electrolyte can realize the phase transition ability of the fluid state and gel state and has a good coating and in situ gelatinization performance, so as to establish the conformal contact and dynamic compliance between electrode and hair scalp. For example, Wang et al. developed a paintable conductive biogel on the skin that shows a temperature-controlled reversible fluid-gel transition [144]. Although dry contact and dry non-contact electrodes are promising for advanced EEG systems, many challenges remain. The contact impedance of the dry electrodes was very high. The development of advanced electrode materials to reduce contact impedance is a strategy to solve this issue. Materials science is developing fast and it will be able to offer more possibilities and contributions. Metal, carbon-based, oxide, and polymer materials show different possibilities for preparing EEG electrodes [18,145,146]. However, future electrodes should not only be made of single materials, but should combine the properties of different materials, such as combining liquid metal with polymer materials, or Mxene with liquid metal and polymer materials, to provide excellent conductivity and flexibility for EEG electrodes. Recently, EEG electrodes are prioritized to use of flexible materials to ensure a certain degree of flexibility and comfort, and more attention is paid to increasing the viscosity of the electrode material to ensure conformality between the electrode and skin to increase the quality of the signal and reduce artifacts [147,148,149]. However, this review suggests that in most practical cases, hair measurement scenarios and good breathability are not negligible. It is also necessary for researchers to consider structural adjustments to ensure conformality and even the rational design of matching electrode caps. For example, intrinsically conductive polymer PEDOT is combined with porous skeleton materials such as a sponge structure to prepare electrodes that can be applied in hair scenes. From our point of view, regardless of the type of EEG electrode, more attention should be paid to the electrolyte phase, electrode material, and structures in order to adapt portable and wearable EEG systems for brain–computer interfaces and medical applications.

## Data Availability

Data sharing not applicable.

## Appendix A

**Table A1:** The formulas involved in the text.

Mathematical Equations		Explanation
Equation (A1)	$SNR=\frac{mean\left(PSD_{band\;of\;interst}\right)}{mean\left(PSD_{signal\;band-band\;of\;interest}\right)}$	*PSD* is the power spectral density. The signal power is defined as the mean *PSD* in the band of interest, whereas the noise power is the mean *PSD* outside this band [98].
Equation (A2)	$r\left(x\right)=\frac{C_{AB}\left(x\right)}{C_{AA}\left(x\right)C_{BB}\left(x\right)}$	where $C_{AB}$ is the cross-covariance between the signals *A* and *B*; $C_{AA}$ is the auto-covariance of the signal *A*; $C_{BB}$ is the auto-covariance of the signal *B*.
Equation (A3)	$Coh\left(x\right)=\frac{{\left|S_{AB}\left(x\right)\right|}^{2}}{S_{AA}\left(x\right)S_{BB}\left(x\right)}$	where $\left|S_{AB}\right|$ is the cross-spectrum between the signals *A* and *B*; $S_{AA}$ is the autospectrum of the signal *A*; $S_{BB}$ is the autospectrum of the signal *B* [5,115,150].
Equation (A4)	$SAR=10\cdot \mathrm{lg}\frac{Energy\left\{EEG_{s}\right\}}{Energy\left\{EOG_{s}\right\}}$	Where $EEG_{s}$ are the *EEG* source signals and $EOG_{s}$ are the *EOG* source signals.

**Table A2:** Comparison of performance of the selected semi-dry EEG electrode.

Electrode	Contact Impedance	Signal Quality		Biocompatibility (Cell Viability)	Ref.
		Stationary	Motion		
Bristle-shaped semi-dry electrode	15 kΩ	Correlation: >90%	-	-	[59]
AgNWs/PVB/melamine sponge (AgPMS)	<10 kΩ	-	-	-	[61]
Flexible multi-layer semi-dry electrode	23.89 ± 7.44 kΩ (Oz) 18.18 ± 7.51 kΩ (Fpz)	Correlation: 95.84%	Correlation: 90.65%	-	[23]
CF-based conductive silicone sponge EEG electrodes	2.5 kΩ	-	-	-	[62]
Micro-seepage electrode	9.3 kΩ (NaCl); 13.5 kΩ (PBS);	-	-	-	[37]
Polymer wick-based quasi-dry electrode	3–5 kΩ	Correlation: 98.2 ± 2.1% Coherence: 97.3 ± 3.0%	-	-	[66]
Passive electrode based on porous Ti	<10 kΩ (NaCl)	Correlation: 95.55% Coherence: 95.2% SNR: 24.4 dB	-	-	[63]
Polycarbonate polymer wick column shape	<30 kΩ		-	-	[67]
Porous ceramics column shape	44.4 ± 16.9 kΩ	Correlation: 88.3−99.7%	-	-	[45,64]
L2-ePt electrode	10.0 ± 6.03 kΩ	Correlation: 94 ± 5.3%	-	-	[65]
Quasi-dry electrode	-	Correlation: 65−97%	-	-	[27]

**Table A3:** Comparison of performance of the selected dry non-contact EEG electrode.

Electrode	Contact Impedance	Signal Quality		Biocompatibility (Cell Viability)	Ref.
		Stationary	Motion		
CNT/aPDMS composite-based dry EEG electrode	<1 MΩ	Coherence: >60% SNR: 3.71 ± 0.17 dB	-	-	[11]
Dry foam-surfaced capacitive electrode	1.5 MΩ hairless 5 MΩ hairy	Correlation: 78.7 ± 2% SNR: 1.88 ± 0.13 dB	-	-	[47]
Novel non-contact dry electrode	-	Correlation: 92.05% Coherence: >80%	-	-	[48]

**Table A4:** Comparison of performance of the selected dry contact EEG electrode.

Electrode	Contact Impedance	Signal Quality		Biocompatibility (Cell Viability)	Ref.
		Stationary	Motion		
On-skin rGO electrodes	>50 kΩ	-	-	-	[69]
Printable flexible Ag/AgCl dry electrode array	19.08–53.65 kΩ	Correlation: 90.8 ± 6.2%	-	-	[70]
Porous Pt ear-EEG electrodes	5 kΩ	-	-	-	[71]
3D-printed dry fingered electrodes	<10 kΩ	SNR: 1.4–3.3 dB	-	-	[72]
Multilayer sweat-absorbable textile electrode	8.97 ± 1.97 kΩ	Correlation: 98.05%	Correlation: 95.6%	-	[73]
GEMMPS	<400 kΩ	-	-	-	[74]
3D-printed dry fingered electrodes	<10 kΩ	Correlation: >98% SNR: 5 dB	-	-	[75]
Nano-modified dry electrode	3.3–19.6 kΩ	Correlation: 81.79–96.77%	-	-	[76]
Ag NWs/PDMS flexible dry electrodes	10 kΩ (no hair) <20 kΩ (hair)	-	-	-	[77,78]
CNF-PDMS	-	Correlation: 90%		-	[116,117]
Multipin dry EEG electrodes	101–102 kΩ	-	-	-	[40]
Dry-contact electrode	435 kΩ	-	-	-	[79]
Soft pin-shaped dry electrode with bristles	101–102 kΩ	Coherence: >90%	-	-	[46]
AgNWs/CNTs/PDMS elastomeric conductive ear electrodes	<102 kΩ	-	-	-	[80]
Graphene electronic tattoo sensors	-	SNR: 7.2 dB	-	-	[118]
Chitosan/Au-TiO2 nanotube-based dry electrodes	67.4 ± 8.9 kΩ	Correlation: 90–95% SNR: <10 dB	-	-	[81]
Flexible silicone-based EEG dry sensor	-	Correlation: 97.85%	-	-	[119]
Dry-contact sensor	<20 kΩ (hair); <10 kΩ (no hair)	Correlation: 98.14%	-	-	[53]
Polyaniline-coated foam electrodes	1.45 MΩ	-	-	-	[82]
Active comb-shaped dry electrodes	102 kΩ	Correlation: 96% SNR: 6.94 dB (thick hair); 7.83 dB (thin hair)	-	-	[83,84]
Multipin shaped electrode	<200 kΩ (forehead); <600 kΩ (occipital)	-	-	-	[41]
Hemispherical claw-like dry electrode	38.6 ± 9.5 kΩ	Correlation: 66 ± 2% SNR: 2.83 ± 2.85 dB	-	-	[86]
Stretchable electrode array	15–60 kΩ	-	-	-	[87]
Dry foam-based EEG	4–12 kΩ	Correlation: 90.12%, 95.56%, 95.12%, 95.82%, 96.14%	-	-	[85,89,93]
Flexible dry PU/TiN-multipin electrodes	72–125 kΩ	-	-	-	[90]
Polymer dry electrodes	<5 MΩ	Correlation: >70% Coherence: >80% SNR: 5.8 dB	-	-	[44]
PDMS-based flexible dry electrode	10 kΩ (no hair) 20 kΩ (hair)	Coherence: 92%	-	-	[91]
Dry-contact electrodes	<150 kΩ	-	-	-	[92]
Multichannel EEG with novel Ti/TiN dry electrodes	250 kΩ–1 MΩ	Correlation: 97.2%		-	[94]
C-electrode	<5 kΩ	-	-	-	[95]
PEDOT-PSS silk electrodes	0.2 kΩ		-	-	[56]
CNT/PDMS-based canal-typed ear electrodes	20 kΩ–1 MΩ	-	-	>93%	[96]
Bristle-sensors	80 kΩ 150–200 kΩ (10 months later)	Coherence: >80%	-	-	[39]
Silver balls based PDMS dry and flexible electrode	10.6 ± 2.7 kΩ	Correlation: 97.2%	-	-	[55]
3D printed dry electrode	10–85 kΩ	-	-	-	[97]
Printed electrodes	-	Correlation: 70.88%	-	-	[121]
Comb-shaped polymer-based dry electrodes	183.5 kΩ–530.0 kΩ	Correlation: 60% Coherence: 34% SNR: 4.3 dB	Correlation: 50% Coherence: 24% SNR: 1.9 dB		[88,98]
Thermally reduced graphene oxide and nylon-membrane sensor	<32 kΩ			95%	[151]
PtSe2 and PtTe2 electronic tattoos	<300 kΩ <10 kΩ (10 KHz)	-	-	PtSe2: >90% PtTe2: >70% (3.125 μ/mL)	[152,153]
MoCl5-intercalated bilayer graphene (Mo-BLG) electrode	-	-	-	88.76 ± 3.35%	[154]

**Table A5:** Comparison of performance of the selected microneedle array EEG electrode.

Electrode	Contact Impedance	Signal Quality		Biocompatibility (Cell Viability)	Ref.
		Stationary	Motion		
LM-based flexible 3D MAE	2.357 ± 0.198 MΩ	-	-	-	[99]
Metal dry bioelectrodes	2.4 kΩ (Ag layer) 1.2 kΩ (Ag–AgCl layer)	-	-	-	[100]
Flexible MAE	<75 kΩ	-	-	-	[25]
Metal dry bioelectrodes	1.181 kΩ	-	-	-	[101]
Flexible P-MNAE with silicon microneedles	64.9 ± 5.4 kΩ	Correlation: 64% Coherence: about 40%	-	-	[102]
MAE	85 kΩ	-	-	-	[51]
Flexible MAE	<50 kΩ	-	-	-	[103]
SU-8 microneedles based dry electrodes	4.2 kΩ (glass); 3 kΩ, 2.7 kΩ, (flexible polyimide);	-	-	-	[104]
Flexible dry electrode	61.2 ± 31.3 kΩ·cm^2^	-	-	-	[105]
Skin screw electrode	<5 kΩ	-	-	-	[106]
Polymer-based dry microneedle electrode	6 kΩ	-	-	-	[107]
Barbed microtip-based electrode arrays	<20 kΩ	Correlation: 91.63%	-	-	[57]
Silicon-based dry electrode	<50 kΩ	-	-	-	[54]
Microneedles-based dry electrodes	13 kΩ	-	-	-	[108]
Microneedles array-based dry electrode	<30 kΩ	-	-	-	[109]
Polymeric microneedle arrays	50 kΩ	-	-	-	[110]
Polymer-based dry microneedle electrode	>200 kΩ <10 kΩ (≥8N)	-	-	-	[52]
A MEMS-based pyramid micro-needle electrode	<8 kΩ	-	-	-	[38]
Micro-spike EEG electrodes	7–25 kΩ	-	-	-	[111]
Microneedle array electrodes	150 kΩ (Ag) 110 kΩ (Ag/AgCl)	Correlation: 95% Coherence: 86% SNR: 24 dB	-	-	[58,112,113]
